# Quantitative Genetics of Growth Rate and Filet Quality Traits in Atlantic Salmon Inferred From a Longitudinal Bayesian Model for the Left-Censored Gaussian Trait Growth Rate

**DOI:** 10.3389/fgene.2020.573265

**Published:** 2020-11-30

**Authors:** Ólafur H. Kristjánsson, Bjarne Gjerde, Jørgen Ødegård, Marie Lillehammer

**Affiliations:** ^1^Stofnfiskur HF, Hafnarfjörður, Iceland; ^2^Department of Animal and Aquacultural Sciences, Norwegian University of Life Sciences, Ås, Norway; ^3^Department of Breeding and Genetics, Nofima AS, Ås, Norway

**Keywords:** Atlantic salmon, growth rate, filet fat, filet pigment, visceral fat, Gibbs sampler, censored

## Abstract

In selective breeding programs for Atlantic salmon, test fish are slaughtered at an average body weight where growth rate and carcass traits as filet fat (*FF*), filet pigment (*FP*) and visceral fat index (*FF*) are recorded. The objective of this study was to obtain estimates of genetic correlations between growth rate (GR), and the three carcass quality traits when fish from the same 206 families (offspring of 120 sires and 206 dams from 2 year-classes) were recorded both at the same age (*SA*) and about the same body weight (*SW*). In the *SW* group, the largest fish were slaughtered at five different slaughter events and the remaining fish at the sixth slaughter event over 6 months. Estimates of genetic parameters for the traits were obtained from a Bayesian multivariate model for (potentially) truncated Gaussian traits through a Gibbs sampler procedure in which phantom *GR* values were obtained for the unslaughtered, and thus censored *SW* group fish at each slaughter event. The heritability estimates for the same trait in each group was similar; about 0.2 for *FF*, 0.15 for *FP* and 0.35 for *VF* and *GR*. The genetic correlation between the same traits in the two groups was high for growth rate (0.91 ± 0.05) visceral index (0.86 ± 0.05), medium for filet fat (0.45 ± 0.17) and low for filet pigment (0.13 ± 0.27). Within the two groups, the genetic correlation between growth rate and filet fat changed from positive (0.59 ± 0.14) for the *SA* group to negative (−0.45 ± 0.17) for the *SW* group, while the genetic correlation between growth rate and filet pigment changed from negative (−0.33 ± 0.22) for the *SA* group to positive (0.62 ± 0.16) for the *SW* group. The genetic correlation of growth rate with *FF* and *FP* is sensitive to whether the latter traits are measured at the same age or the same body weight. The results indicate that selection for increased growth rate is not expected to have a detrimental effect on the quality traits if increased growth potential is realized through a reduced production time.

## Introduction

Growth rate (*GR*) is among the most important traits selected for in selective breeding programs for Atlantic salmon. Improved growth rate enables faster turnover in production, and this creates economic benefits in terms of reduced fixed and variable costs per kg fish produced. The increased growth rate is expected to reduce the fraction of the nutrient in the feed consumed that is allocated to maintenance and hence, improving feed efficiency. Improved feed efficiency was detected in a farmed salmon population selected for increased growth rate over five generations when compared to wild salmon (Thodesen et al., 1999). Over generations, genetic improvement of growth rate will result in cohorts of fish reaching the appropriate body weight at a younger age, resulting in shorter production time. Therefore, the growth rate to targeted body weight (*GR*_*SW*_) rather than a targeted age (*GR*_*SA*_) is likely the most appropriate breeding objective trait for growth.

Other important breeding objective traits are filet (carcass) fat (*FF*), visceral fat (*VF*) and filet pigment (*FP*). For FF and VF the breeding goal may be to keep or reduce their trait level since increasing body fat could potentially increase feed conversion ratio (*FCR*)as shown in a study of rainbow trout (Kause et al., 2016). Unfortunately, estimates of the effect of selection for reduced *FF*, *VF* or increased *FP* on feed efficiency, or the correlated effect in feed efficiency through selection for other traits (e.g., growth), is not possible to obtain as feed consumed by fullsib families is not possible to obtain on a sufficiently large number of families at an affordable cost. And currently, no tools or equipment are available to obtain individual feed consumption records of fish reared in a group. Breeding goal of *FP* is to increase redness of the filet since consumers are not as willing to buy a pale salmon filet (Steine et al., 2005).

In current breeding programs for Atlantic salmon, the traits mentioned above *GR*,*FF*,*VF* and *FP* are recorded when the average body weight of the test fish group(s) reach a targeted round body weight similar to typical commercial slaughter weight (e.g., 4–5 kg), at which point all fish are slaughtered over a few days, and therefore approximately at the same age (*GR*_*SA*_,*FF*_*SA*_,*FP*_*SA*_,*VF*_*SA*_), or over a few slaughter events to reduce biomass without any particular grading with respect to body weight. The recording of the traits is therefore not performed at a specific body weight in line with the ideal definition in the breeding objective (*GR*_*SW*_,*FF*_*SW*_,*FP*_*SW*_,*VF*_*SW*_) as the fastest and the slowest growing fish will, respectively, be well above and well below the targeted weight. Consequently, there is a discrepancy between the recorded traits and their definition in the breeding goal. The main reason for this is that recording the traits at about the same body weight is labor-demanding and also stressful for the fish, as the fish need to be graded frequently so that the appropriate fraction of the largest fish can be slaughtered and measured at each grading event. For fish reared under natural environmental conditions, e.g., in floating net cages in the sea in which the seawater temperature and daylight vary over the year, introducing sample slaughter would also introduce substantial environmental differences and handling stress between the fish at the different slaughter events which may cause biased estimates of parameters and breeding values.

In Atlantic salmon estimates of genetic correlations between *GR*_*SA*_ and *FF*_*SA*_ are relatively high (0.34–0.74) (see Appendix 2). If these positive correlations reflect the corresponding genetic correlation between growth rate (*GR*_*SW*_) and filet fat (*FF*_*SW*_), simultaneous genetic improvement of the two traits may be difficult to achieve. To reduce the impact of this seemingly unfavorable genetic correlation, estimated breeding values for *FF*_*SA*_ maybe obtained by including body size of the fish as a covariate in the statistical model, or by pre-correcting the *FF*_*SA*_ records for body size. This would account for both environmental and genetic effects of body size on *FF*_*SA*_ and may therefore affect both the genetic and residual correlations of *FF*_*SA*_ with *GR*_*SA*_ and other traits. This was illustrated in two studies in Atlantic salmon where the genetic correlation between body weight (*GR*_*SA*_) and filet fat (*FF*_*SA*_) changed from positive to negative when *FF*_*SA*_ was accounted for body weight (from 0.45 to −0.22 (Rye and Gjerde, 1996) and from 0.45 to −0.10 (Vieira et al., 2007)). This illustrates the importance of having reliable estimates of the genetic correlation between the traits as defined in the breeding objective as this may have large effects on both the predicted responses of the traits under selection, the predicted correlated responses in other traits and on the relative weighting needed to obtain the desired gain in each of the traits.

For fish slaughtered at the same age estimates of genetic correlation between *GR* and *FF* are also found to be positive in Coho salmon, Arctic char, common carp, and sea bream, but negative in rainbow trout and close to zero in European whitefish (see Appendix 2). Between *GR* and *FP* both positive and negative correlations are reported, while negative correlations seem to be the most common of *FF* with *FP* and *VF*. For the magnitude of the few other genetic correlations reported in Appendix 2 (those between *GR* and *VF* and between *FP* and *VF*) no clear picture can be drawn.

The objective of this study was to obtain reliable genetic parameter estimates for *GR*_*SW*_, *FF*_*SW*_, *VF*_*SW*_ and *FP*_*SW*_ by sampling and recording the traits at about the same body weight (*SW*). For comparison, the traits were also recorded on a different sample of sibs from the same families when slaughtered at the same age (*GR*_*SA*_, *FF*_*SA*_, *VF*_*SA*_ and *FP*_*SA*_). The *SW* and the *SA* fish were reared in tanks at a land-based facility in which seawater temperature and natural light over the experimental period to provide as similar environmental conditions as possible for the *SW* fish slaughtered at the six different slaughter events.

## Materials and Methods

On request, authorities in Iceland stated that the recording of body weights of live fish does not require a special permit. The two other traits were recorded on dead fish. All fish was kept and managed according to Icelandic law.

### Fish and Their Rearing

The Atlantic salmon in this study were from the breeding nucleus of Stofnfiskur in Iceland. The material used consisted of 2 year-classes produced in fall 2008 (yc 1) and spring 2009 (yc 2) using a nested mating design where each female was mated to one male and each male to two females in most cases, but some males were mated with a single female only. Within each year class, all matings were completed over 4 weeks. Year-class (yc) 1 consisted of 106 fullsib families (offspring of 106 females and 68 males) and yc 2 of 100 families (offspring of 100 females and 52 males). From fertilization until start feeding the families were reared in separate hatching trays at Stofnfiskur family unit. The yc 1 families were startfed over a 11 days period from 20/4/2009 to 1/5/2009, while the families in yc 2 were startfed over 12 days from 10/11/2009 to 22/11/2009. From startfeeding until individual tagging of the fish, the families were reared separately in 1.5 m^2^ tanks at Stofnfiskur family unit. At an average body weight of 15 g, a random sample of 100 fish from each fullsib family were individually tagged with PIT (Passive Integrated Transponder) tags deposited into the abdomen cavity of the fish. After tagging the fish were reared in a common tank until smoltification at an average body weight of 80 g. After smoltification, the tagged smolt of each year class was transported and reared in a common on-shore and in-door tank at Stofnfiskur breeding stations in Kalmanstjörn (yc 1) or Vogavík (yc 2). Rearing was under natural light and using borehole seawater with natural and stable salinity (ranging from 30 to 31‰ Kalmanstjörn and from 23 to 28‰ Vogavík) and temperature (ranging from 10 to 11°C in Kalmanstjörn and from 7.5 to 9°C in Vogavík). Genetic correlations between growth rate until an average body weight of 3 kg at these two farms have repeatedly found to be high (Jónas Jónasson pers comm.) and thus negligible genotype by environment interaction for growth. The feed used was commercial feed pellets containing 25% fat (22.9 MJ/kg) and 50 mg astaxanthin/kg (Vörur, 2020). The fish received ad-lib feeding adjusted to appetite.

### Two Experimental Groups

The fish of each year-class were reared in one (yc 1) and four (yc 2) tank(s) until an average body weight of 2.5 kg, at which the fish of each year-class and family were divided randomly into two groups, one slaughtered at the same age (*SA*) and the other at about the same body weight (*SW*). All the *SA* group fish were slaughtered when they reached the average target body weight of about 4.6 kg, while the *SW* group fish were slaughtered at an individual target body weight of about 4.6 kg and thus at different ages.

For yc 1 the group sizes were 10 and 13 individuals per family for the *SA* and *SW* group, respectively; while for yc 2, the group sizes for both groups (*SA* and *SW*) were 15 individuals per family.

### Slaughtering of the SA Group

The *SA* groups of both year-classes were reared in one tank from an average body weight of 2.5 kg to the desired harvest body weight and were harvested over 5–7 days; yc 1 889 to 904 days from first feeding (9335 to 9492°d) at an average body weight of 4.4 kg with a standard deviation of 1.1 kg, and yc 2 1024 to 1038 days from first feeding (8448 to 8564°d) at an average body weight of 4.6 kg with a standard deviation of 1.3 kg.

### Sampling and Slaughtering of the SW Group

The *SW* yc 1 was reared in two tanks from an average body weight of 2.5 kg. After the third sampling from each of the two tanks, the biomass was sufficiently reduced to pool the fish into one tank (see Table 1). The *SW* yc 2 was reared in one tank from an average body weight of 2.7 kg until the end of the experiment.

**TABLE 1:** Descriptive statistics of the studied traits for each year-class and experimental group of the SW group at each sampling and slaughter date.

			Age		Body weight,kg		Growth rate, g/day			Filet fat, %		Filet pigment, mg/kg		Visceral Index		Body weight, kg		Growth rate, g/day	
												
Slaughter nr.	Sample	Date	Days	*N*	Mean	SD	Mean	SD	*N*	Mean	SD	Mean	SD	Mean	SD	Mean	SD	Mean	SD
**Year-class 1**																			
0	ST	07.03.2011	700	1276	2.44	0.64	3.49	0.91											
	RA	03.05.2011	748	93	2.48	0.68	3.31	0.91											
	RA	21.06.2011	797	92	3.39	0.90	4.26	1.13											
1	SL	05.07.2011	805	250	4.41	0.52	5.48	0.65	167	13.38	0.98	7.12	0.53	9.30	1.28	4.67	0.43	5.80	0.53
2	SL	09.08.2011	840	481	4.31	0.39	5.13	0.47	215	13.72	0.88	7.66	0.56	7.76	1.77	4.65	0.24	5.54	0.28
	RA	23.08.2011	860	101	4.15	0.70	4.82	0.82											
3	SL	30.08.2011	861	449	4.44	0.39	5.15	0.45	257	14.17	0.96	7.41	0.92	7.43	1.38	4.71	0.21	5.47	0.24
	RA	16.09.2011	884	97	4.55	0.64	5.15	0.72											
4	SL	04.10.2011	887	579	4.23	0.70	4.77	0.79	290	14.90	1.23	6.91	0.66	7.17	1.15	4.77	0.26	5.38	0.29
	RA	16.10.2011	911	54	4.13	0.71	4.53	0.79											
5	SL	01.11.2011	923	268	4.55	0.51	4.92	0.55	143	15.80	1.05	7.27	0.60	7.37	1.28	4.82	0.30	5.19	0.28
6	SL	30.11.2011	958	156	4.05	0.79	4.22	0.82	156	14.50	1.47	6.98	0.72	7.45	1.28	4.05	0.78	4.22	0.82
**Year – class 2**																			
0	SL	05.12.2011	1079	1418	2.73	0.77	2.53	0.71											
1	SL	31.05.2012	1142	349	3.75	1.11	3.28	0.97	131	15.51	1.03	7.94	0.51	6.26	0.87	4.85	0.62	4.26	0.54
2	SL	17.07.2012	1190	233	4.87	0.34	4.10	0.28	203	16.24	1.12	8.24	0.67	6.41	0.94	4.87	0.34	4.10	0.29
	RA	22.08.2012	1225	94	3.95	0.88	3.22	0.72											
3	SL	31.08.2012	1234	359	4.68	0.47	3.79	0.38	268	16.44	1.27	7.88	0.87	6.48	10.1	4.87	0.34	3.95	0.28
	RA	20.09.2012	1254	98	4.08	0.86	3.25	0.68											
4	SL	01.10.2012	1265	486	4.54	0.44	3.59	0.35	308	15.98	1.16	7.31	0.59	6.13	0.89	4.81	0.28	3.80	0.22
5	SL	31.10.2012	1295	282	4.35	0.37	3.36	0.29	143	16.72	1.39	6.83	0.66	6.79	1.03	4.64	0.17	3.59	0.13
6	SL	05.12.2012	1330	333	4.05	0.83	3.04	0.62	333	15.48	1.79	6.77	0.57	6.73	1.06	4.05	0.82	3.04	0.62

*Sample abbreviations as follows: SD,standard deviation; ST,start when sorting the group for the trial; RA,random sample; SL,slaughter.*

In both year-classes, a fraction of the largest fish was slaughtered at five different slaughter events and the remaining fish at a sixth slaughter event over 148 (yc 1) and 188 (yc 2) days, and with 167 to 290 fish (yc 1) and 131 to 333 fish (yc 2) being slaughtered at each slaughter event (Table 1). The number of days between each slaughter event varied from 21 to 35 (yc 1) and from 30 to 47 (yc 2) days.

At the first slaughter event for both year classes, fish larger than 4.2 kg were slaughtered, while for the four following slaughtering events fish larger than 4.4 kg were slaughtered. In this way, the average targeted body weight of 4.6 kg (4.65 to 4.82 g in yc 1 and 4.64 to 4.87 kg in yc 2) was obtained for the five first slaughtering events. At the sixth and last slaughter event, the average body weight of the remaining fish was 4.05 kg in both year classes.

The fish to be slaughtered were sampled and kept in a separate tank for 1 week until being slaughtered by cutting the gills and bled before fileting. At each of these samplings, the body weight of some fish just below the set body weight threshold for slaughter were also recorded since the fish were subjectively sampled. These fish were not slaughtered at the actual slaughter event. The number of fish with body weight records just below the set threshold can be found as the difference between the number of recorded and slaughtered fish in Table 1. For yc 1 this number of fish was 83, 266, 192, 289, and 125, for slaughter event 1, 2, 3, 4, and 5, respectively; and similarly, for yc 2 218, 30, 91, 178, and 139 fish.

The body weights of the fish of a few random samples (five in yc 1 and two in yc 2) were obtained 4–6 days before some of the slaughter events, primarily to find the appropriate time for each slaughtering, but also to investigate if including or omitting these records from the statistical analyses have an effect on the parameter estimates. The number of individuals and dates of measure are given in Table 1.

All the sampled fish were anesthetized by manually picking up the fish from the tank and placing it into a 200-liter container with 100 ml of Phenoxyethanol.

**FIGURE 1:**
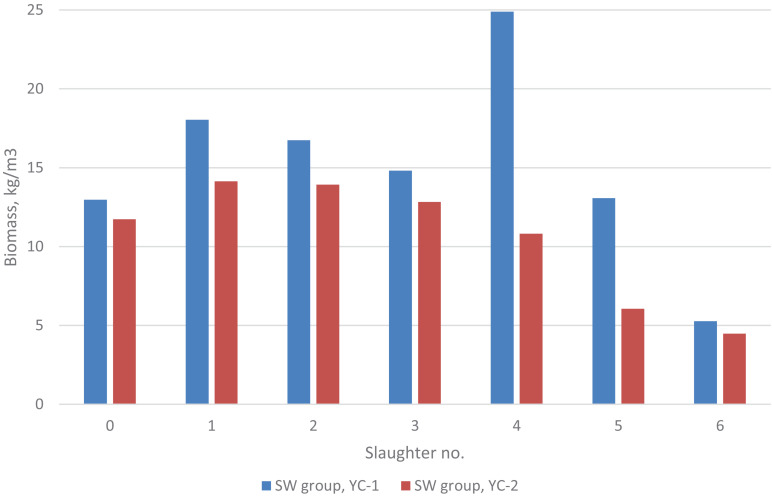
Biomass for the SW group at the start (0) and at each of the six slaughter events of the 2 year-classes. For the SA group, the biomass at slaughter was 18 kg/m^3^ (yc 1) and 27 kg/m^3^ (yc 2).

For the *SW* group, the biomass (kg/m^3^ seawater in the rearing tank) over the experimental period is shown in Figure 1. For yc 1 it was 13 at first recording and 18, 17, 15, 25 (two tanks merged into one tank), 13 and 5 kg/m^3^ at each of the six slaughtering events, respectively; while for yc 2 it was 12 at first recording and 14, 14, 13, 11, 6, and 5 kg/m^3^ at each of the six slaughtering events, respectively. Similarly, for yc 1 the fish density (no of fish/m^3^) was 5.3 at first recording and 4.6, 3.7, 2.7, 1.5, 0.9, and 0.2 at each of the six slaughtering events, respectively; while for yc 2 it was 4.3 at first recording and 3.9, 3.3, 2.5, 1.5, 1.1, and 0.1 at each of the six slaughtering events, respectively. For the *SA* group, the biomass at slaughter was 18 kg/m^3^ (yc 1) and 27 kg/m^3^ (yc 2).

### Traits Recorded

For both the *SW* and the *SA* groups the following traits were recorded at slaughter for each of the 2 year classes: the round body weight (*BW* in kg), filet fat (*FF*_*SW*_, *FF*_*SA*_, in %), filet pigment (*FP*_*SW*_, *FP*_*SA*_, in mg/kg), and visceral weight (including liver, gut and intestinal fat) divided by the round body weight to obtain visceral index (*VF*_*SW*_, *VF*_*SA*_ in %) as an indicator of visceral fat (Kause et al., 2007). For the *SW* group the body weight (*BW*, in kg) of all fish were recorded when the average body weight of the whole group was 2.4 kg (yc 1) and 2.7 kg (yc 2). Growth rate (*GR*_*SW*_, *GR*_*SA*_, in g/day) was calculated as round body weight divided by the number of days from the first feeding to slaughter.

Filet fat (*FF*_*SA*_,*FF*_*SW*_) and filet pigment (*FP*_*SA*_, *FP*_*SW*_) were measured on both filets in pre-rigor state.*FF* was predicted based on backscatter of light in the near-infrared spectra (NIR, wavelengths at 15 channels between 760 and 1040 nm). *FP* was predicted based on backscatter of visible light (VIS, wavelengths at 15 channels between 430 to 730 nm) the visual (VIS) spectra using the Qmonitor (TOMRA, 2020) installed at Stofnfiskur, Iceland (see next paragraph). These wavelength spectra were used as the explanatory (and predictor) variables, while the response variables were the chemically analyzed filet fat and filet pigment values of a homogenized sample of the whole filet without skin as the response variables (Folkestad et al., 2008). The average predicted filet fat and filet pigment value of both filets were used.

### Prediction Model for Filet Fat and Filet Pigment

The prediction model for filet fat and filet pigment was developed based on data obtained from a sample of 24 Atlantic salmon weighing between 1 to 6 kg. The fish were from the same breeding nucleus population as the experimental groups (see section “Fish and Their Rearing”). The mean filet fat of the fish was 13.7% (standard deviation 2.1% units), and the mean filet pigment was 7.4 mg astaxanthin (standard deviation 1.4 mg/kg).

The prediction models were developed using *PLS* (Partial Least Squares) regression (Tormod Næs, 2002). Prediction error was reduced further by Canonical Partial Least Squares (CPLS) regression (Indahl et al., 2009) where additional information from each fish was included (round body weight, filet weight and visceral weight).

As the variation in the fat content within a filet is very high a better prediction model for filet fat, than using the average fat value of the filet, was obtained by using the fat content of five selected filet plug samples from each filet (a total of 120 plugs, each of approximately 15 mm in diameter) as the response variables and the NIR wavelengths spectra from the same locations as the plugs as the explanatory variables (Segtnan et al., 2009).

The fat content of each of the 120 plugs was obtained from a low-field nuclear magnetic resonance (H-NMR) instrument (Marin Ultra, 23 MHz, Oxford Instruments, United Kingdom) at Nofima, Ås and which are highly correlated to chemical analyzed fat values (Sørland et al., 2004).

The remaining part of each filet without skin was minced using a food blender, and a 30 g sample was analyzed for fat (%) (Soxhlet method), astaxanthin (mg/kg) and canthaxanthin (mg/kg) at Nofima, Sunndalsøra. The prediction model for filet fat of the whole filet was validated using the chemically analyzed fat values of 24 filets (one filet from each fish).

The summary statistics for the prediction models for filet fat and filet pigment in this study and the filet fat model developed in Segtnan et al. (2009) where the plug sampling methodology was described are shown in Appendix 1. For filet fat in the whole filet, the *PLS* based prediction model had a root mean square error of prediction (RMSEP) of 2.02%-units as compared to 1.88%-unit for the *CPLS* model. For filet pigment, the *RMSEP* was 0.84 mg/kg using PLS regression and did not improve when using *CPLS* regression.

### Statistical Methods

In the *SW* group, the faster-growing fish were slaughtered before the slower-growing fish. Hence, *BW*_*SW*_ and its corresponding trait value *GR*_*SW*_ were truncated trait values recorded at six different time points over the 6 months experimental period, but with only one record per fish for most of the fish. Therefore, as the fish at each time point were slaughtered at about the same body weight, mean *GR*_*SW*_ at each time point will decrease over time. Consequently, if only the sampled fish were included in the analysis at each time point, the parameter estimates for *GR*_*SW*_ and other traits (*FF*_*SW*_, *FP*_*SW*_, *VF*_*SW*_) would be biased.

Hence, a statistical model was needed which accounted for the body weight distribution of all fish present at each of the six sampling events. The available data for such a model was the *BW*_*SW*_, *FF*_*SW*_, *VF*_*SW*_ and *FP*_*SW*_ records of the fish slaughtered at each of the six slaughter events, the body weight records of the sampled but not slaughtered fish, and the (ID of) remaining fish in the tank(s) at each slaughter event and known to be smaller than any of the slaughtered fish.

For this purpose, a Bayesian multivariate model for (potentially) truncated Gaussian traits (Ødegård et al., 2010) implemented in the Gibbs sampling module in DMU (Jensen et al., 2014) was used. The procedure simulates left-censored growth rate phenotypes for the fish with no *GR*_*SW*_ records at each of the six slaughter events, sampled from a truncated normal distribution, upwardly truncated at the set body weight threshold.

**FIGURE 2:**
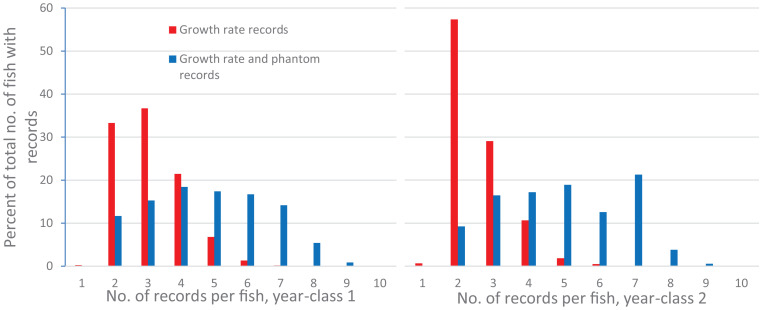
Percent of the total number of fish with growth records with 1 or up to 10 repeated growth records, or 1 or up to 10 growth and phantom records for each of the 2 year-classes.

Estimates of (co)variances for the random effects and BLUE-estimates for the different levels of the fixed effects for the studied traits were obtained from a multi-trait animal model with eight traits (*GR*_*SW*_, *FF*_*SW*_, *FP*_*SW*_, *VF*_*SW*_, *GR*_*SA*_, *FF*_*SA*_, *FP*_*SA*_ and *VF*_*SA*_). *GR*_*SW*_ was a left-censored trait (including a few recorded but not slaughtered individuals below the threshold) with at least two and up to ten records per fish (Figure 2).

Yc 1 and yc 2 were first analyzed separately. Estimated (co)variances for the traits were similar and did not differ significantly between the 2 year-classes. Therefore, the datasets from both year-classes were analyzed jointly. In matrix notation the model may be written as:

(1) $$Y=\left[\begin{array}{c} Y_{1-7}\\ Y_{8} \end{array}\right]=Xb+Za+Mc+\left[\begin{array}{c} 0\\ S \end{array}\right]r+e$$

The vector **Y_1–7_** represented the seven traits *GR*_*SA*_, *FF*_*SA*_, *FP*_*SA*_, *VF*_*SA*_, *FF*_*SW*_, *FP*_*SW*_, *VF*_*SW*_ with only one record per animal which was not censored since they were not subject to selection, while the vector **Y_8_** represented the trait *GR*_*SW*_ which was a left-censored longitudinal trait with two to ten repeated *GR*_*SW*_ records (including the censored phenotypes). For trait ***Y*_1–7_** the fixed effects included the combination of year-class (2 year-classes), tank (seven tanks) and sex (males and females). The fixed effects for ***Y*_8_** were year-class, tank, sampling group (23 groups) and sex; the vector **a**∼**N**(**0**,**A**⊗**G**_**0**_) included the additive animal genetic effects for each of the studied traits where ***A*** was the numerator relationship matrix constructed from the pedigree of the parents and grandparents and **G_**0**_** was the additive genetic (co)variance matrix; the vector **c**∼**N**(**0**,**I**⊗**C_**0**_**) included the effects common to fullsibs other than additive genetics and **C_**0**_** was the (co)variance matrix of effects common to full-sibs; the vector $\text{r}∼\text{N}\,\left(0,\text{I}\,\sigma_{r}^{2}\right)$ included the individual repeatability effects due to two or more repeated *GR*_*SW*_ records on the same fish; **e**∼**N**(**0**,**I**⊗**R_**0**_**) was a vector of random residuals and **R_**0**_** was the residual (co)variance matrix.

For each of the 2-year classes, the *SA* and *SW* traits were recorded on different individuals, resulting in independent residuals between traits in the *SA* and the *SW*groups, and thus **e**∼**N**(**0**,**R_**0**_**), where:

$$R_{0}=\left[\begin{array}{ccc} R_{0_{1-4}} & 0 & 0\\ 0 & R_{0_{5-7}} & 0\\ 0 & 0 & I{\text{σ}}_{e_{8}}^{2} \end{array}\right]$$

where **R**_**01**−**4**_was the residual (co)variance matrix of the four traits in the *SA* group, **R**_**05**−**7**_ was the residual (co)variance matrix for the traits *FF*_*SW*_, *FP*_*SW*_, *VF*_*SW*_in the *SW* group and $\sigma_{e_{8}}^{2}$ was the residual variance of *GR*_*SW*_. *GR*_*SW*_ was a longitudinal trait, while all other traits were cross-sectional. Hence, this method did not allow residual correlations between *GR*_*SW*_ and other traits in the *SW* group to be estimated. However, the advantage of longitudinal modeling of *GR*_*SW*_ was that it accounts for the non-random slaughter of the fish at each of the six slaughtering events.

The matrices **X**, **Z** and **M,** are incidence matrices that assign the observations to their appropriate fixed effect, random additive genetic and common fullsib effects, respectively. The matrix **S** assigns the phenotypes of repeatability effect to the trait *GR*_*SW*_ (not relevant for the other traits). For an individual *I* still alive at time point *j* with body weight below the sampling threshold, the growth rate phenotype was drawn from the truncated normal distribution (TN) as:

$${\text{Y}}_{8,i\,j}∼\text{T}\,N\,\left({\text{X}}_{8\,i}\,\text{b}+{\text{Z}}_{8\,i}\,\text{a}+{\text{M}}_{8\,i}\,\text{c}+{\text{S}}_{i}\,\text{r},\sigma_{e_{8}}^{2},-\infty ,\frac{{\text{TW}}_{j}}{{\text{t}}_{i\,j}}\right)$$

where the growth phenotype was truncated in the interval − to $\frac{{\mathrm{TW}}_{j}}{t_{i\,j}}$, where *TW_j* was the threshold weight at time ***j*** (the body weight of the smallest slaughtered fish) and **t**_**i***j*_ was the age (days from start feeding) for fish ***i*** at time ***j***. The TN distribution has also fixed and random effects for individual ***i***.

The model was run for 2.017.200 rounds, discarding the first 10.000 samples as burn-in, with a sample interval of 100 rounds; thus the estimated (co)variances were based on 20.072 rounds retained from the Monte Carlo Markov Chain (MCMC) chain. Convergence was evaluated using Raftery and Lewis convergence diagnostics (Raftery and Lewis, 1992) using the package Coda (Plummer et al., 2018) in the statistical program R (R Development Core Team, 2018). Raftery and Lewis reveal how many rounds from the MCMC are needed by evaluating 2.5% quantile from the chain at given precision with the probability 0.95. If the precision was set to 0.02, the desired number of rounds was lower than 20.072 for all parameters. If the precision was set to 0.1 the following parameters $\sigma_{F\,F\,S\,A}^{2},{\text{h}}_{F\,F\,S\,A}^{2},{\text{h}}_{F\,P\,S\,W}^{2},{\text{r}}_{G\,R\,S\,A,F\,P\,S\,A},{\text{r}}_{G\,R\,S\,A,F\,F\,S\,W}$ needed more rounds.

Heritability **h**^2^ was calculated as the additive variance $\sigma_{a}^{2}$ divided by the phenotypic variance $\sigma_{p}^{2}$ denoted as

$${\text{h}}^{2}=\frac{\sigma_{a}^{2}}{\sigma_{p}^{2}}$$

Where $\sigma_{p}^{2}=\sigma_{a}^{2}+\sigma_{c}^{2}+\sigma_{e}^{2}$***;*** $\sigma_{a}^{2}$ was the additive genetic variance, $\sigma_{c}^{2}$ was the variance of the effect common to fullsibs, and $\sigma_{e}^{2}$ was the residual variance. For the trait *GR*_*SW*_ the $\sigma_{p}^{2}$ also contains the repeatability variance $\sigma_{r}^{2}$ so the phenotypic variance becomes.

$$\sigma_{p}^{2}=\sigma_{a}^{2}+\sigma_{c}^{2}+\sigma_{e}^{2}+\sigma_{r}^{2}$$

The proportion of the variation due to the effect common to fullsibs ***c*^2^** was calculated as the variance common to fullsibs $\sigma_{c}^{2}$ divided by the phenotypic variance $\sigma_{p}^{2}$ defined as

$${\text{c}}^{2}=\frac{\sigma_{c}^{2}}{\sigma_{p}^{2}}$$

The genetic correlation between trait 1 and 2 (**r**_**g1**,**2**_), the correlation of the effect common to fullsibs between trait 1 and 2 (**r**_**c1**,**2**_**)**, and the residual correlation between trait 1 and 2 (**r**_**e1**,**2**_**)** were calculated as

$${\text{r}}_{g\,1,2}=\frac{\sigma_{g\,12}^{2}}{\sigma_{g\,1}\,\sigma_{g\,2}}\;{\text{r}}_{c\,1,2}=\frac{\sigma_{c\,12}^{2}}{\sigma_{c\,1}\,\sigma_{c\,2}}\;{\text{r}}_{e\,1,2}=\frac{\sigma_{e\,12}^{2}}{\sigma_{e\,1}\,\sigma_{e\,2}}$$

### Effects of Pre-correcting *FF*_**S***A*_ for Body Weight

It is of interest to investigate if traits recorded at the same age (*SA*) can be adjusted to obtain parameter estimates comparable to those obtained for trait recorded at the same body weight (*BW*). In this paper, we limit this to a small investigation for the trait *FF* with a pre-correction of the observed *FF*_*SA*_ trait values for their corresponding *BW*_*SA*_ records. An in-depth study of how to best perform this will be the objective of another paper.

First, the regression coefficient of *FF*_*SW*_ on *BW*_*SW*_ was obtained from the following linear model, separately for each of the 2 year-classes:

(2) $$F\,F_{S\,A}=\beta_{0}+\beta_{1}\,B\,W_{S\,A}+e$$

This regression coefficient (β_1_) was used to generate the pre-corrected phenotype *preFF*_*SA*_ as follows, for each of the 2 year-classes:

(3) $$p\,r\,e\,F\,F_{S\,A}=F\,F_{S\,A}-\beta_{1}\,B\,W_{S\,A}$$

The genetic correlation of *preFF*_*SA*_ with *FF*_*SW*_,*GR*_*SW*_ and *GR*_*SA*_ were obtained from bivariate animal models with the same fixed effect as in Model 1.

## Results

### Descriptive Statistics

The total number of slaughtered individuals with records for all the studied traits were 1228 (yc 1) and 1386 (yc 2) for the *SW* group and 965 (yc 1) and 1412 (yc 2) for the *SA* group. In addition, there were 48 (yc 1) and 32 (yc 2) fish with growth records that died before reaching the targeted body weight for slaughter. The percentage of fish in the *SW* group lost due to mortality, and typographical errors were 4.2% (yc 1) and 7.6% (yc 2) of the total number of fish at the start (*ST*) of the sampling (see Table 2). For the *SA* group, the corresponding numbers were 2.1% (yc 1) and 1.2% (yc 2).

**TABLE 2:** Descriptive statistics for the four studied traits of each year-class and experimental group^1^.

		Growth, g/day			Visceral index, %			Filet pigment, mg/kg			Filet fat, %		
Year-class	Group	*N*	Mean	CV × 100	*N*	Mean	CV × 100	*N*	Mean	CV × 100	*N*	Mean	CV × 100
1	SA	961	4.96	23.4	964	6.13	18.3	965	7.29	11.8	965	13.79	11.5
1	SW	3904	4.43	24.2	1276	7.66	19.8	1260	7.22	10.2	1228	14.39	9.2
2	SA	1412	4.47	28.4	1414	5.28	15.9	1412	7.53	11.3	1412	17.29	12.8
2	SW	3647	3.31	24.8	1418	6.46	15.5	1385	7.44	11.6	1386	16.02	8.9

*^1^For the SA group, the mean round body weight (CV × 100) at slaughter was 4.40 kg (23.4) for yc 1 and 4.60 kg (28.3) for yc 2.*

The descriptive statistics of the four studied traits in Table 2 show that the mean observed growth rate of yc 1 was higher than of yc 2 for both the *SA* and the *SW* group, probably because yc 1 was reared at a higher water temperature than yc 2 (see section “Fish and Their Rearing”). For visceral index and filet fat, some differences in mean values were observed between the *SA* and the *SW* groups, within and across the 2 year-classes, but with no clear trend. Average filet fat was higher in yc 2 than in yc 1 for both the *SA* (3.5%-units higher) and the *SW* (1.6%-units higher) group. For the *SA* group this may be due to the about 200 g higher mean body weight of yc 2 (4.60 kg, CV 28.3%) than of yc 1 (4.40 kg, CV 23.4%), while for *SW* the overall mean body weight of the slaughtered fish was 4.61 kg for yc 1 and 4.68 for yc 2 with a CV 8.0% for yc 1 and 9.1% for yc 2. Mean values for filet pigment were very similar for the two groups within and across the 2 year-classes.

Furthermore, Table 2 shows that the coefficient of variation (*CV*) of growth rate was similar for the *SA* and the *SW*groups. Very similar *CV* for the two groups was also observed for visceral index and filet pigment of each year-class, while for filet fat a somewhat higher *CV*was found for the *SA* group than for the *SW* group. For filet pigment means and *CV* for the *SA* and *SW*groups were very similar within and across the 2 year-classes.

Table 1 shows that the mean body weight of the *SW*group at the five first slaughtering events ranged from 4.65 to 4.82 kg (yc 1) and from 4.64 to 4.87 kg (yc 2), and thus close to the set desired body weight of 4.6 kg. The *CV* of body weight at each slaughtering event varied from 4.5 to 9.0% (yc 1) and from 3.6 to 12.8% (yc 2) for slaughter events one to five. The mean body weight of the fish slaughtered at the sixth and last slaughtering event was lower (4.05 kg for both year-classes) as all the remaining fish were slaughtered at this slaughter event and therefore with a larger *CV* (19.3% for yc 1 and 20.4% for yc 2) than for the fish slaughtered at the five first slaughtering events. *CV* of filet fat varied from 6.6 to 8.3% (yc 1) and from 6.7 to 8.3% (yc 2) for slaughter event one to five but was higher at the sixth and last slaughter events (*CV* 10.1% for yc 1 and 11.5% for yc 2) most likely due to the larger variation in body weight. For each year-class, the filet pigment was quite similar over the six slaughter events and with quite similar standard deviations and thus different *CV*s (*CV* 6 to 12%), while the visceral index at each of the six slaughter events had similar standard deviations but different means and thus different *CVs* (*CV* 16 to 26%).

For the *SW* group, the mean observed filet fat percentage increased throughout the slaughter events while the mean observed growth rate decreased (Table 1). This indicates that slow growers add more fat in the filet than fast growers but could also be interpreted as filet fat generally increases with age.

For yc1 there were in total 3904 growth records and 6139 growth and phantom records, and for yc 2 3647 growth records and 6963 growth and phantom records. Of the total number of fish with growth records 91.5% (yc 1) and 97.0% (yc 2) had two to four repeated growth records (Figure 2), while 45.4% (yc 1) and 42.9% had two to four growth and phantom records (Figure 2).

### Observed and Estimated Growth Rate at Each Slaughter Event of the SW Group

**FIGURE 3:**
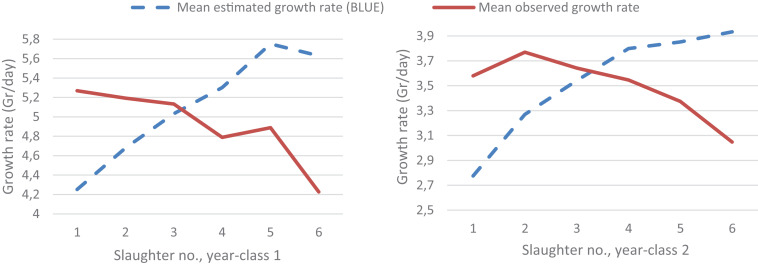
The mean observed growth rate of all the recorded fish (the slaughtered ones and those close to the set body weight threshold for slaughtering) and the BLUE-estimates for the mean growth rate of all fish (the recorded ones and the not recorded fish that were still alive in the tank) at each of the six slaughter events for each of the 2 year-classes.

In Figure 3, the decreasing mean observed growth rate over the six slaughter events showed that the fastest-growing fish were slaughtered first. The difference between the mean observed and the mean estimated growth rate is due to the slaughter and body weight recording of only the largest fish at each slaughter event, which the statistical model is meant to account for through assigning phantom growth rate phenotypes for the fish with no body weight record at each of the five first slaughter events. The estimated growth curve is expected to equal the growth curve that would be realized if the body weight of all or a random sample of the fish (i.e., not selected on body size) was recorded at each slaughter event.

### Heritability

Table 3 shows that the estimated heritability for the same trait in the two groups was quite similar whether recorded at the same age (*SA*) or the same body weight (*SW*); of medium magnitude (0.20–0.37) for *GR*, *FF* and *VF*, but lower for *FP* (0.11–0.16).

**TABLE 3:** Estimates of heritability of the studied traits (on the diagonal) and genetic (below the diagonal) and residual (above the diagonal) correlations between the traits based on the data from both year classes.

	*FF*_*SA*_	*FP*_*SA*_	*VF*_*SA*_	*GR*_*SA*_	*FF*_*SW*_	*FP*_*SW*_	*VF*_*SW*_	*GR*_*SW*_
*FF*_*SA*_	0.23 ± 0.08	−0.35 ± 0.03	0.04 ± 0.05	0.69 ± 0.03	−	−	−	−
*FP*_*SA*_	−0.37 ± 0.23	0.11 ± 0.04	−0.08 ± 0.04	−0.16 ± 0.04	−	−	−	−
*VF*_*SA*_	−0.12 ± 0.19	0.08 ± 0.20	0.37 ± 0.06	−0.21 ± 0.05	−	−	−	−
*GR*_*SA*_	0.59 ± 0.14	−0.33 ± 0.22	−0.13 ± 0.16	0.33 ± 0.08	−	−	−	−
*FF*_*SW*_	0.45 ± 0.17	−0.03 ± 0.23	−0.17 ± 0.15	−0.35 ± 0.18	0.20 ± 0.04	−0.20 ± 0.03	−0.05 ± 0.03	0.41 ± 0.02
*FP*_*SW*_	0.26 ± 0.24	0.13 ± 0.27	0.09 ± 0.19	0.58 ± 0.17	−0.38 ± 0.20	0.16 ± 0.05	−0.13 ± 0.04	−0.01 ± 0.03
*VF*_*SW*_	−0.14 ± 0.20	0.06 ± 0.20	0.86 ± 0.05	0.14 ± 0.17	−0.45 ± 0.13	0.16 ± 0.20	0.35 ± 0.06	−0.06 ± 0.03
*GR*_*SW*_	0.44 ± 0.18	−0.31 ± 0.23	−0.09 ± 0.16	0.91 ± 0.05	−0.45 ± 0.17	0.62 ± 0.16	0.19 ± 0.17	0.35 ± 0.09

### Genetic and Residual Correlations

Estimates of genetic and residual correlations among the traits are given in Table 3. The genetic correlation between the same trait in the two groups was high for *GR* (0.91 ± 0.05) and *VF*(0.86 ± 0.05) indicating that these traits are not sensitive to whether recorded at the same age (*SA*) or the same body weight (*SW*), and will thus result in quite similar ranking of the families whether recorded at *SW* or *SA*. For *FF*, the genetic correlation was of medium magnitude (0.45 ± 0.17) and rather low for *FP* (0.13 ± 0.27) which implies substantial reranking of families for each of these traits when recorded at *SW* or *SA*.

Within each of the two groups, the genetic correlation between *GR* and *FF* changed from positive (0.59 ± 0.14) for the *SA* group to negative (−0.45 ± 0.17) for the *SW* group, while the genetic correlation between *GR* and *FP* changed from negative (−0.33 ± 0.22) for the *SA* group to positive (0.62 ± 0.16) for the *SW* group. Similarly, the genetic correlation between *GR* and *VF* was not significantly different from zero but changed from slightly negative (−0.13 ± 0.16) for the *SA* group to slightly positive (0.19 ± 0.17) for the *SW* group. Within both groups, the genetic correlation of *FF* with *FP* and *VF* was medium to low negative but not significantly different from zero, while those between *FF* and *VF* were low but positive but also not significantly different from zero.

The residual correlations between *FF*, *FP* and *VF* within each of the two experimental groups were low, while that between *GR* and *FF* was relatively high in the *SA* group (0.69 ± 0.03) and somewhat lower in the *SW* group (0.41 ± 0.02).

The low residual correlation between *FF* and *FP*, in both the *SA* (−0.35) and the *SW* (−0.20) groups, shows that these traits to a large extent were independently predicted. Most likely this is because the *FP* and *FF* values were obtained based on two different VIS and NIR wavelength spectra, respectively; and that the response variable in the prediction model for *FP* was the chemical analyzed pigment and not the visual filet color.

### Effect Common to Fullsibs

Table 4 shows that the effect common to fullsib as a proportion of the phenotypic variance was rather low, being highest for *GR*_*SW*_(0.14 ± 0.04), *FF*_*SA*_ (0.12 ± 0.04) and *GR*_*SA*_ (0.12 ± 0.04).

**TABLE 4:** The effect common to fullsib as a proportion of the phenotypic variance (on the diagonal) and the correlation between the trait for this effect.

	*FF*_*SA*_	*FP*_*SA*_	*VF*_*SA*_	*GR*_*SA*_	*FF*_*SW*_	*FP*_*SW*_	*VF*_*SW*_	*GR*_*SW*_
*FF*_*SA*_	0.12 ± 0.04	−	−	−	−	−	−	−
*FP*_*SA*_	−0.59 ± 0.17	0.07 ± 0.02	−	−	−	−	−	−
*VF*_*SA*_	0.21 ± 0.28	−0.19 ± 0.27	0.06 ± 0.03	−	−	−	−	−
*GR*_*SA*_	0.77 ± 0.12	−0.37 ± 0.23	0.17 ± 0.28	0.12 ± 0.04	−	−	−	−
*FF*_*SW*_	0.50 ± 0.22	−0.36 ± 0.26	−0.07 ± 0.31	−0.03 ± 0.28	0.05 ± 0.02	−	−	−
*FP*_*SW*_	0.12 ± 0.23	0.31 ± 0.22	0.06 ± 0.29	0.39 ± 0.20	−0.34 ± 0.24	0.08 ± 0.03	−	−
*VF*_*SW*_	0.16 ± 0.28	−0.12 ± 0.27	0.70 ± 0.18	0.33 ± 0.28	−0.28 ± 0.28	0.06 ± 0.28	0.06 ± 0.03	−
*GR*_*SW*_	0.65 ± 0.16	−0.34 ± 0.22	0.21 ± 0.27	0.87 ± 0.08	−0.11 ± 0.27	0.48 ± 0.19	0.38 ± 0.27	0.14 ± 0.05

The fullsib (family) correlations between the same trait in the two groups were positive (Table 4). The correlations between different traits within the *SA* and *SW* groups (Table 4) were similar except for *FF* and *GR* which changed from strongly positive (0.78 ± 0.11) in the *SA* group to close to zero within the *SW* group (−0.09 ± 0.27). The correlation between *FP* and *GR* changed from negative in *SA* (−0.37 ± 0.22) to positive in *SW* (0.47 ± 0.20). Therefore, the fullsib effect correlations between these traits seem to be sensitive to whether phenotypes are recorded at the same age or about the same body weight.

### Pre-correction of the Quality Traits

The genetic correlation between *preFF*_*SA*_ and *GR*_*SA*_was 0.05 ± 0.18 as compared to the much higher genetic correlation of 0.69 ± 0.03 between *FF*_*SA*_ and *GR*_*SA*_ and the much lower genetic correlation of −0.45 ± 0.17 between *FF*_*SW*_ and *GR*_*SW*_. In addition, the genetic correlation between *preFF*_*SA*_ and *FF*_*SW*_ was 0.81 ± 0.09 as compared to the much lower genetic correlation of 0.45 ± 0.17 between *FF*_*SA*_ and *FF*_*SW*_. Consequently, pre-correction of the *FF*_*SA*_ records for body weight brought the genetic correlation between *FF* and *GR* recorded at the same age closer to the genetic correlation between the same two traits when recorded at the about the same body weight.

## Discussion

### Genetic Parameters

The objective of this study was to obtain reliable genetic parameters of growth rate (*GR*), filet fat (*FF*), visceral fat (*VF*) and filet pigment (*FP*) when these traits were recorded on fish slaughtered at about the same body weights (*SW*) and varying age, and compare these with the parameter estimates of the same traits when recorded on their sibs at the same age (*SA*) and thus at different body weights. The heritability of each trait recorded at *SW* and *SA* were similar. However, the genetic correlations between the same trait in the *SA* and *SW* groups were moderate for *FF* (0.45±0.17) and low for *FP* (0.13±0.27). Also, some of the genetic correlation estimates changed sign whether recorded at *SW* or *SA*; between *GR* and *FF* 0.59 ± 0.14 for *SA* vs. −0.45 ± 0.17 for *SW*, between *GR* and *FP* −0.33 ± 0.22 for *SA* vs. 0.62 ± 0.16 for *SW*, and between *GR* and *VF* −0.13 ± 0.16 for *SA* vs. 0.19 ± 0.17 for *SW*. As the parameter estimates were consistent across the 2 year-classes, these results strongly suggest that *FF* and *FP* should be viewed as different traits and will cause substantial reranking of families when tested both at *SA* and *SW*. The moderately positive genetic correlation between *GR* and *FF* recorded at the same age (*SA*) agree well with published results for Atlantic salmon as well as for several other farmed fish species (see Appendix 2).

The low Genetic correlation between filet fat recorded at *SA* and *SW* and filet pigment recorded at *SA* and *SW*, strongly indicate that if these traits are directly selected for in a breeding program, the time of their recording (*SA* or *SW*) is highly relevant. As growth rate is an important trait in all selective breeding programs, selection for increased growth rate will likely result in commercially farmed fish being slaughtered at younger ages with each successive generation, potentially also altering the mean phenotypes and the genetics of the quality traits at the time of slaughter. This may complicate efficient selection for carcass quality traits. If selection is practiced for increased growth rate only, the genetic correlations of growth rate with the quality traits obtained at *SW* reveals likely their correlated effect when the fish are slaughtered at about the same body weight.

The relatively high genetic correlation between *GR*_*SA*_ and *GR*_*SW*_ (0.91 ± 0.05) indicates that growth rate is largely the same trait whether recorded at *SW* or *SA*. For growth the genetic correlation between body weights measured on the same animals at different ages and thus different body weights were found to be high when measured near in time (within a few months), but lower when measured further apart (Gjerde et al., 1994; Powell et al., 2008), indicating that growth should be measured at body weight as defined in the breeding objective.

### Importance and Breeding Objective of Quality Traits in Atlantic Salmon

Production of an Atlantic salmon with more body fat than required from a marketing point of view should be avoided as deposition of fat requires more energy than deposition of protein (Knap and Kause, 2018), and as a fatty fish is likely also to be more costly to produce depending on the relative price of the fat and protein feed ingredients. A theoretical calculation shows that if the body fat of a salmon can be reduced by 1%-unit, the energy need of the fish could be reduced by about 0.4 MJ/kg, corresponding to a 0.034 reduction in *FCR* for a feed with 24.2 MJ/kg (T. Åsgård, pers. Comm), which for the Norwegian salmon industry (1.4 billion tons in 2019) amounts to about 50 000 tons of feed.

The breeding objective for *FF* depends foremost of the desired filet fat level in the most important salmon market(s), at what body size the fish are and will be harvested in the future as *FF* increases with body weight, and the present genetic potential for *FF* deposition of the animals in the actual breeding nucleus population. Given that selection for increased *GR* will result in an earlier harvest of fish at about the same body weight, it may be concluded that due to the negative genetic correlation between *GR*_*SW*_ and *FF*_*SW*_ (−0.45 ± 0.17), as well as between *GR*_*SA*_ and *FF*_*SW*_ (−0.35 ± 0.18), selection for increased *GR* is more likely to give a favorable correlated response in *FF* (i.e., a reduction) than the opposite. Consequently, *FF* may not need to be recorded or selected for unless the filet fat level becomes too low. However, by recording *FF* it becomes possible to reduce *FF* faster than possible through a correlated response through selection for increased *GR*, which may also be favorable from a feed efficiency trait point of view (Kause et al., 2016).

*VF* must be considered as a waste product but should not be reduced to a level with a negative effect on the fitness of the fish. For instance, reduced *VF* may affect reproduction as *VF* (and *FF*) is mobilized during sexual maturation (Aksnes et al., 1986), and the effect on reproduction may become larger if *FF* is also reduced. The low negative genetic correlation between *GR*_*SW*_ and *VF*_*SW*_ (0.19 ± 0.17) indicates that selection for increased *GR* will result in a modest but unfavorable correlated response in *VF*. Consequently, to obtain a reduction in *VF* will require *VF* being recorded so that directional selection against this trait can be applied.

The most likely breeding objective for *FP* is to increase the retention efficiency of the carotenoids in the feed, and thus allow for the production of a fish with sufficiently high *FP* using a cheaper feed with less carotenoids, or for a more pigmented filet to obtain a higher price (Steine et al., 2005; Alfnes et al., 2006). However, during the last years, the economic value of *FP* has been reduced as costs associated with pigment in the feed has been reduced substantially and accounts for only 1.1–3.6% of the feed costs (Cargill) as compared to 15%, 15 years ago (Steine et al., 2005; Alfnes et al., 2006). Also the relatively high genetic correlation between *GR*_*SW*_ and *FP*_*SW*_ (0.62 ± 0.16) strongly indicates that selection for increased growth rate will result in a favorable correlated response in *FP* and also with a low but most likely favorable genetic correlation of *FP*_*SW*_ with both *FF*_*SW*_ and *VF*_*SW*_.

### Reliability of the Parameter Estimates

An important assumption for the above discussion is that the parameter estimates for the traits recorded at *SW* are both unbiased and accurate; i.e., that the Gibbs sampling procedure managed to account for the selection and recording of only the largest fish at five of the six slaughter events, and that the number of recorded fish at each event is sufficiently high to allow the Gibbs procedure to work properly.

The purpose of harvesting only the largest fish at five of the six slaughter events was to obtain the three carcass quality trait records at a body weight which is more in line with the most likely breeding objective of these traits, i.e., at about the same body weight, as compared to recording the traits at the same age as is the practice in today’s selective breeding programs. The mean observed and estimated growth rate at each slaughter event (Figure 3) indicate that the Gibbs sampling procedure, to a large extent, managed to account for the culling on body weight. This is also supported by the fact that excluding the body weight (i.e., the *GR*_*SW*_) records of the approximately 100 fish randomly sampled prior to slaughter event 1, 3, 4 and 5 (yc 1) and 3 and 4 (yc 2) (see Table 1) changed the parameter estimates only marginally. The effect of this culling for body weight on the quality traits cannot be accounted for in the same manner as for *GR*_*SW*_ but only through their correlation to *GR*_*SW*_. Consequently, *GR*_*SW*_ is the only trait that can be modeled as a censored trait, and with only the overall mean as a fixed effect in the model for each of the quality traits. Therefore, for each of the quality traits a figure similar to Figure 3 for *GR*_*SW*_ is not possible to produce. To what degree the correlations of growth trait with the quality traits are sufficient to produce unbiased parameter estimates for the latter traits can only be inferred using stochastic simulation where the true genetic (co)variances among the traits are known.

The unbiasedness of the estimated parameters for the traits of the *SW* group may be affected by changes in the rearing conditions (e.g., water temperature, feed, biomass and fish density) over the six slaughter events as these may have an effect on what degree culling with respect to body weight was properly accounted for through the Gibbs sampling procedure (see section “Rearing Conditions”). The accuracy of the estimated parameters for these traits depends on the number of slaughter events and the number and proportion of the fish slaughtered at each event. Moreover, since each fish in the *SW* group had at least two growth records, a repeatability effect could be estimated for the *GR*_*SW*_ trait, while no such effect could be estimated for the quality traits in the *SW* group. Given this, residual covariance of *GR*_*SW*_ with each of the three quality traits are difficult to estimate since *GR*_*SW*_ has many residuals per fish while each of the quality traits has only one. Hence, the residual term for *GR*_*SW*_ should be interpreted differently than for the other traits in the *SW* group. The unbiasedness and accuracy of the parameters can only be inferred from a well-designed stochastic simulation study where the true parameters are known.

### Rearing Conditions

The fish in the *SA* group of each of the 2 year-classes were all slaughtered at the same time, and thus influenced by the same environmental rearing conditions until being slaughtered and the traits recorded. This is in contrast to the fish in the *SW* group for which the trait records were obtained at six different slaughter events over 6 months and thus being influenced by varying rearing conditions that may have had a different effect on each of the recorded traits. If these environmental effects were not properly accounted for by the Gibbs sampling procedure, this might have resulted in biased parameters. In this study water temperature and salinity was very stable over the entire experimental period, type of feed was the same and feed was given according to the predicted biomass over time. However, both biomass (kg/m^3^) and fish density (no. of fish/m^3^) varied over the six slaughter events with a possible effect on the growth as well as on the quality traits of the *SW* fish. These possible effects cannot be accounted for *per se* in the present data or using other data sources due to a lack of such published effects on the traits. In most studies where the effect of tank size and fish density on growth is evaluated, larger tanks and lower densities result in better growth (Refstie and Kittelsen, 1976; Espmark et al., 2017). Having a low number of fish in a tank can revel strong social hierarchies with effect on growth (Ranta and Pirhonen, 2006) and with a possible effect on the growth rates in particular the two last slaughter events. The effect of changes in the rearing environment on the growth of the fish in the *SW* group was sought to be accounted for by including the starting point and the six slaughter events (first column of Table 1) as a fixed effect in the statistical model (which also accounts for the age of the fish which may impact both their body composition and growth). Due to the relatively stable rearing conditions in the present study, we are confident that the Gibbs sampling procedure to a large extent managed to account for the relatively strong culling for body weight at five of the six slaughter events as well as for the relatively minor changes in environmental conditions over the experimental period. Performing a similar experiment, e.g., in a net-cage in the sea in which the fish are exposed to a much larger change in the water temperature with a strong effect on growth rate would probably have resulted in less reliable parameters for traits in the *SW* group.

### Recording the Quality Traits at SA or SW

Recording carcass quality traits at the same age of the fish is much less labor demanding than recording them at about the same body weight. However, the latter procedure is more in line with how quality traits should ideally be defined in the breeding objective. Therefore, if some carcass quality traits are to be directly selected for in a selective breeding program the question that remains to be answered is whether genetic parameters and breeding values for traits recorded on fish at the same age or about the same body weight are comparable.

In some breeding programs, an adjustment of the quality trait records for body weight is performed, e.g., by including body weight as a covariate for each quality trait, or by pre-correcting their phenotypes as exemplified for filet fat in chapter 2.8. These results strongly indicate that pre-correction of filet fat for body weight brings the genetic correlation between *preFF*_*SA*_ with *GR*_*SW*_ closer to the genetic correlation between *FF*_*SW*_ and *GR*_*SW*_, and that pre-correcting the *FF*_*SA*_ records for body weight can be a practical way to obtain a good predictor for *FF*_*SW*_ more in line with how the traits most likely should be defined in the breeding objective. However, adjusting a trait for another genetically correlated breeding objective trait may affect the genetic and residual variances of the adjusted trait and its genetic and residual correlation to other traits. Only if the adjusted trait and the correlated trait have equal heritability and equal genetic and residual correlation, the two traits are genetically independent. This has been shown for feed intake adjusted for a production trait, but apply to any other trait that is defined as a linear function of another trait (Kennedy et al., 1993). To what degree *FF*_*SA*_ will be adjusted also for its genetic relationship to *GR*_*SA*_ is therefore dependent of the magnitude of both the genetic and residual (co)variances of the traits, and consequently in most cases with an unknown and maybe also non-wanted effect on the relative genetic gain of the traits.

The pre-correction of *FF*_*SA*_ also revealed that *preFF*_*SA*_ is a trait more similar to *FF*_*SW*_ as inferred from the much higher genetic correlation between pre*FF*_*SA*_ and *FF*_*SW*_ (0.81) than between *FF*_*SA*_ and *FF*_*SW*_ (0.45). This indicates that the purpose of recording quality traits at *SW* rather than at *SA* is mainly to obtain reliable genetic correlations that are more in line with their most likely definition in the breeding objective.

How to perform a simultaneous selection for increased growth rate and reduced body fat is also an important issue in livestock species. However, literature addressing how to treat high unfavorable genetic correlations between traits is limited. High genetic correlations have been detected between body weight and intramuscular fat when the traits were measured at the same age; e.g., 0.71–0.84 in broilers (Zerehdaran et al., 2004) and 0.87 in Texel sheep (Clelland et al., 2014). In fattening pigs a high genetic correlation is also found between growth rate and carcass fat growth, both measured from 25 to 100 kg live weight, and thus slaughtered at about the same body weight (0.84 in Landrace, 0.72 in Duroc), while the genetic correlation between growth rate and muscle (lean) growth during the same period was close to zero (−0.06 in Landrace, 0.07 in Duroc) (Gjerlaug-Enger et al., 2012). Based on the findings in this study, an alternative for terrestrial animals species could be to measure the carcass quality traits at about the same body weight and thus over a period of time. Then apply Gibbs threshold model to correct the body weight records for the selection performed for growth rate at the time of recording the quality traits and thus obtain predicted quality traits records less dependent on body size.

## Conclusion

The estimated genetic correlations of growth rate with filet fat, filet pigment and visceral index were found to be sensitive to whether the traits were recorded at the same age or about the same body weight. In commercial production, increased genetic growth potential is expected to be realized through reduced production time and thus slaughtering the fish at a younger age. Hence, genetic correlations between growth rate and carcass quality traits recorded at about the same body weight are likely more relevant than those recorded at the same age. The result indicates that selection for increased growth rate is not expected to have a detrimental effect on the studied carcass quality traits given that the increased growth potential is realized through a reduced production time.

## Data Availability Statement

The raw data supporting the conclusions of this article will be made available by the authors, without undue reservation.

## Ethics Statement

Ethical review and approval was not required for the animal study because On request authorities on Iceland stated that obtaining body weights on live fish does not require a special permit. The other traits were recorded on dead fish. Written informed consent was obtained from the owners for the participation of their animals in this study.

## Author Contributions

BG and JØ designed the study. ÓK, BG, ML, and JØ carried out the statistical analysis and interpreted and discussed the results. ÓK conducted the experiments and wrote a first draft of the manuscript in close cooperation with BG and ML. BG, ML, and JØ reviewed and approved the final manuscript. All authors contributed to the article and approved the submitted version.

## Conflict of Interest

ÓK was employed by the company Stofnfiskur HF. The remaining authors where employed by the offical reaserach institude Nofima. Stofnfiskur pays for the supervision of ÓK at Nofima, but the remaining authors declare that the research was conducted in the absence of any commercial or financial relationships that could be construed as a potential conflict of interest.

## Appendix

**APPENDIX 1:** Summary statistics of available prediction models for filet fat and filet pigment, using NIR (Near Infrared) and VIS (Visual) reflectance spectroscopy measures andby means of PLS (partial least squares) orCPLS (canonical partial least squares) regression.

Model developer	Dependent variable	Type of tissue	No. of records	Prediction model method	RMSEP	*R*^2^
Segtnan et al. (2009)	Filet fat	Plugs	145	PLS	1.96%-units	0.90
Kristjánsson (2012)	Filet fat	Plugs	120	PLS	2.02%-units	0.88
Kristjánsson (2012)	Filet fat	Plugs	120	CPLS	1.88%-units	0.90
Kristjánsson (2012)	Filet fat	Whole filet	24	CPLS	0.39%-units	0.99
Kristjánsson (2012)	Filetpigment	Whole filet	24	PLS	0.84 mg/kg	0.82

*RMSEPis the Root Mean SquaredError of Prediction) and *R*^2^ is the coefficient of determination of the model.*

**APPENDIX 2:** Estimates of published genetic correlations of growth rate (GR) with filet fat (FF), filet pigment (FP) and visceral fat (VF), and of FF with FP and VF; when these traits were all measured at the same age (SA) in several farmed fish species.

r_G*R*,F*F*_	r_G*R*,F*P*_	r_G*R*,V*F*_	r_F*F*,F*P*_	r_F*F*,V*F*_	Species	References
0.42	0.31	−0.64	−0.82	−0.67	Atlantic salmon	Rye and Gjerde (1996)
0.45	0.2		0.00		Atlantic salmon	Vieira et al. (2007)
0.34–0.75	−0.41–−0.19		−0.3		Atlantic salmon	Powell et al. (2008)
0.84	−0.17		−0.19		Atlantic salmon	Tsai et al. (2015)
−0.19	0.21	0.19	−0.44	−0.33	Rainbow trout	Gjerde and Schaeffer (1989)
−0.12	0.36	0.38	0.13	−0.43	Rainbow trout	Kause et al. (2002)
0.24–0.36	0.50–0.73		−0.67–0.02		Coho salmon	Iwamoto et al. (1990)
0.73					Coho salmon	Neira et al. (2004)
	0.15–0.25				Coho salmon	Dufflocq et al. (2017)
0.82	0.65		0.22	0.91	Arctic charr	Elvingson and Nilsson (1994)
0.59	−0.61				Arctic charr	Wolters et al. (2013)
0.59					Common carb	Kocour et al. (2007)
−0.08			0.55		European white fish	Kause et al. (2011)
0.29					Sea bream	García-Celdrán et al. (2015)
0.59	−0.33	−0.13	−0.37	−0.12	Atlantic salmon	Current study
